# Eliminating trichiasis: the next steps forward

**Published:** 2012

**Authors:** Paul Courtright, Matthew Burton, Paul Emerson

**Affiliations:** Co-Director, Kilimanjaro Centre for Community Ophthalmology International, www.kcco.net; Senior Lecturer, International Centre for Eye Health, London School of Hygiene and Tropical Medicine; Director, Trachoma Control Programme, The Carter Center, Email: Paul.Emerson@emory.edu

Significant progress is being made in the fight against blinding trachoma. However, according to estimates, there are over 7.3 million people with trachomatous trichiasis (TT) in the world.^1^

Trachomatous trichiasis occurs when in-turned eyelashes scrape the cornea. Not only is this incredibly painful, it also causes damage to the cornea; sufferers will become irreversibly blind unless it is corrected surgically.

Providing access to quality surgical services, based in the community, is critical if we are to reach the target of eliminating blindness due to trachoma.

Since the formation of the Global Alliance for the Elimination of Blinding Trachoma (GET2020) in 1997, there have been advances in trichiasis management on many fronts. However, surgical numbers remain quite low.

The Global Trichiasis Scientific Meeting was held in Tanzania earlier this year to consider evidence from various sources about how best to manage trichiasis. The meeting was attended by scientists, national ministry of health programme managers, and NGO personnel from ten countries affected by trachoma. Here are selected conclusions from the meeting that are particularly relevant to eye care professionals actively working in the field:

## Surgical management

- It is both necessary and possible to improve trichiasis surgery. Programmes and surgeons should follow the techniques outlined in the WHO manual “Final Assessment of Trichiasis Surgeons”^2^
- The bilamellar tarsal rotation procedure can produce excellent results.
- Specific guidelines for managing recurrent TT are limited or lacking in most settings. Programmes are encouraged to develop locally appropriate guidelines for the management of recurrent TT.
- Evidence indicates there is no difference in the TT recurrence rates between silk and synthetic absorbable sutures.
  **Figure:**
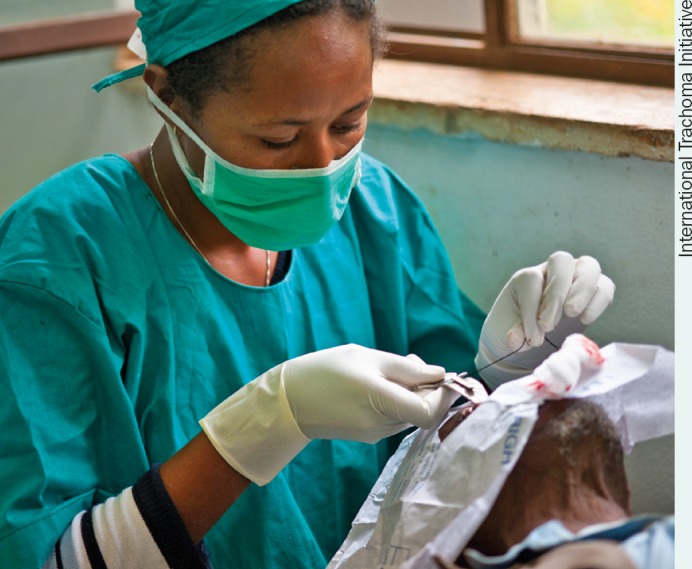
Eyelid surgery for trichiasis. ETHIOPIA
- Definite indications for surgery include: having any central lashes, having peripheral lashes that touch the cornea, or surgery requested by the patient with TT. There is consensus that even a few lashes touching the peripheral globe (peripheral TT) should be managed with an appropriate intervention.
- Even minor peripheral TT can progress and cause corneal damage; epilation can be considered but this must be accompanied with appropriate counselling and follow-up.
- Pre-operative TT should be stratified and recorded as: minor: 0–4 lashes, major: 5–20 lashes, and severe: 20+ lashes. This will make follow-up easier.
- Post-operative TT can be subdivided into early or late, and into clinically significant and non-significant. Clinically significant post-operative TT includes: any central lashes, peripheral lashes that touch the cornea, and TT identified as significant by the patient. Ideally, initial post-operative follow-up should take place between six and twelve weeks, and before six months.

## Surgical training and quality

- The WHO Final Assessment of Trichiasis Surgeons manual should be adopted by all programmes and incorporated at all levels of training.
- There is programmatic evidence that the trainees being put forward for TT surgery training are not always the most suitable candidates. Programmes should adopt and follow clear criteria for trainee selection.
- There isa need fora training of trainers manual. Where possible, training curricula should be standardised at the national level to ensure that different training programmes have equivalent outcomes.
- Supportive supervision should be built into the programmes from the beginning. Surgery team leaders should be selected from amongst active TT surgeons and receive additional training and resources to ensure they are empowered to do their job.
- Surgeons should maintain a surgical register of all patients. Auditing of clinical outcomes should be part of ongoing supervision. There should be a first year audit after training, including a review of patient cards and productivity, and some review of patient outcomes and observation of surgery.

## Surgical output and uptake

- Static service delivery alone will not solve the problem; outreach (bringing the service to the patient, or the patient to the service) is needed to address the backlog.
- Evidence suggests that in well-planned, well-organised, and effectively managed programmes, surgeons can each operate on 20 eyes per day.
- Decisions on who to train as TT surgeons will be country specific. A significant proportion of general health workers who are trained as trichiasis surgeons later stop performing operations; this requires attention from the country's ministry of health.
- Findings in multiple settings suggest that programmes to encourage people to come for surgery should have an understanding of the barriers that a community faces. In some settings, where use of services is lower among women, gender-focused strategies are needed.
- There is sufficient evidence that bringing the service to the patient increases uptake; however, even with community-based trichiasis surgery, it is unlikely that a programme will achieve 100% coverage and interventions are needed to manage those who refuse surgery.

Activities to address some of the challenges are underway; these will be reported in future articles in this series. The meeting was sponsored by the Fred Hollows Foundation and Lions SightFirst.
